# Third Day’s Proceedings

**Published:** 1863-07

**Authors:** 


### THIRD DAY'S PROCEEDINGS.

The Association convened at 9 o'clock. After a partial reading of the minutes, the further reading was dispensed with.

The following gentlemen were admitted permanent members by invitation:—Isaac Snyder, Mich.; R. B. Treat, Janesville, Wis.

The following were admitted as permanent members:—Granville S. Thomas, Joliet, Ill.; J. S. Pashley, Osceola, Ill.

Dr. Cox, of the army, announced the sudden departure of Dr. Wilson Jewell, of Pennsylvania, caused by receiving intelligence of the unexpected death of a son, and offered a resolution of condolence which was adopted.

Regular business being in order, the reports of Committees were taken up.

The President having announced that the order of the Surgeon-General U. S. A., debarring calomel and tartar emetic from the use of army surgeons, and which was previously referred to a Committee, was in order, by consent of the Association the Committee on the subject offered a substitute for the resolutions introduced yesterday.

Pending the discussion, previous to the vote, Dr. Cox, of the army, said substantially as follows:

While the Association had the right to protest against the order of the Surgeon General, he wished it to remember that the order referred exclusively to the corps of army surgeons under his control, and had no reference to the use of those drugs in private practice. The order originated in the abuse of calomel by a number of incompetent surgeons in the army, appointed by the Governors of the several States, who consider the *liver* the pack-horse of the human system. The Medical Bureau of the United States comprises men of science, who understand how far the evil has been perpetuated and the necessity of correcting its abuses. The fact that other mercurials have not been interfered with, shows how great the necessity that exists for an order so apparently sweeping, and which the Association deems so arbitrary.

He did not desire to protract the debate, but felt it due his position to say something before the final vote should be taken. He was not up either to defend or condemn the order. In a long practice he had seen the abuse of calomel in improper hands, as well as its benefits from its legitimate and judicious use. He wished a discrimination to be made between the propriety of the order of the Surgeon General. That gentleman's high character and motives are not to be questioned in this or any other public body. He deserved the thanks of the profession for the wholesome interest he had taken in the subject.

Dr. Cox's position called up several members in reply. Calomel had fallen under the ban of an "unwise, unnecessary, and unprofessional" order, and that order received animadversion, ridicule and unstinted opposition. The discussion became general, and while some desired to place no obstacle in the field, their opinion of the order was of a character that culminated in the following resolutions:

*Resolved.* That from evidence now in our possession, we can but entertain the conviction that the Surgeon General of the U. S. Army has been led into expressions, in order No. 6, which will convey errors respecting the abuse of calomel in the army, and we feel called upon to protect, so far as

Page(s) missing

11. The increase of the heart's impulse and the dyspnœa in patients with organic cardiac diseases disappeared even in cases in which digitalis had done nothing.

12. After the normal temperature of the body has been raised by the use of iron, it lasted a considerable time after stopping with its use before returning to its normal condition whilst the morbid lowered temperature rose quickly by the use of iron it fell just as quickly by stopping with its use—at least, where the other pathological symptoms continued, and where consequently the cause of the low temperature was not cured.

Referring to these facts, the Doctor lays down the following maxims:—Taking into consideration that the temperature of the body and the quantity of urea in the urine is increased by the use of iron, that the œdematous condition disappears and the weight of the body is augmented, we are fully justified in ascribing to the iron a nutritive power. The increase of temperature indicates a stronger tissue-change, for this is constant, and accompanied by other symptoms indicating a heightened nutrition. How this is brought about it is difficult to say. Increase of the blood quantum or of the blood corpuscles cannot be the cause; both increase very slowly, whilst the change of tissue augments very quickly. Neither can the increase of the pulse explain the elevated temperature, as the first succeeds the latter. The respiration is not altered by the iron, hence cannot have an influence upon the temperature.

According to Dr. Pakrowsky, we have, therefore, to look for the effect of iron in the finest arterial and capillary system, one of the most important places of nutrition, and the growth of the tissue and organs, and so much more, as the disappearance of dropsical transudations in the subcutaneous cellular tissue after the use of iron, points to that system. The most probable is the supposition that the iron acts upon the contractile elements of the finest arterial branches, which must have, without doubt, a high and important influence upon the capillary circulation, and, namely, upon the degree of the tonics, i. e. the degree of tension of the walls of these ramifications. The iron must consequently alter the conditions of the diffusion of the elements composing the tissue and organs. Only in this way does it seem possible to explain the quick effect of iron upon nutrition and the resorption and the œdematous transudations.—*Cincinnati Lancet and Observer.*

